# When Cancer Meets Dementia: The End-of-Life Caregiving Experience for Older Adults With Comorbid Dementia and Cancer

**DOI:** 10.1093/geroni/igaa057.1529

**Published:** 2020-12-16

**Authors:** Gigi C C Ling, Amy Y M Chow

**Affiliations:** 1 The Chinese University of Hong Kong, Hong Kong, China; 2 The University of Hong Kong, Hong Kong, China

## Abstract

Older adults with comorbid dementia and cancer is an increasing phenomenon with the aging population worldwide. Caregivers of these older adults might have a totally different and unique end-of-life caregiving experience. This is because all physical and behavioral signs and symptoms of dementia and cancer may interact with each other and complicate the caregiving experience. The aims of this study was to understand and examine the end-of-life caregiving experiences for older adults with comorbid dementia and terminal cancer from the perspective of family caregivers. Twenty-one caregivers were invited to participate in a semi-structured interview that examined the end-of-life caregiving experiences, its impact and how they coped with the challenges they faced. The interviews were transcribed and analyzed using interpretative phenomenological analysis. The essential meaning of the phenomenon is understood as “grieving thrice, suffering dually and becoming one”, characterized by how caregivers understood the meaning of togetherness after going through the time of recurring losses from dementia through cancer to death and experiencing ambiguous sufferings dually with their loved one. Ambiguous sufferings were not “there” before the diagnosis of cancer but emerge in the context of comorbid dementia and cancer and in the connection with the caregivers making interpretation and appraisal of their internal and external resources. These important findings fill in the knowledge gap in the literature related to end-of-life caregiving experience for older adults with comorbid dementia and cancer; and may guide the development of appropriate interventions to support the older adults and their caregivers in a holistic approach.

